# Design of Prodrugs to Enhance Colonic Absorption by Increasing Lipophilicity and Blocking Ionization

**DOI:** 10.3390/ph7020207

**Published:** 2014-02-24

**Authors:** Rebecca Nofsinger, Sophie-Dorothee Clas, Rosa I. Sanchez, Abbas Walji, Kimberly Manser, Becky Nissley, Jaume Balsells, Amrithraj Nair, Qun Dang, David Jonathan Bennett, Michael Hafey, Junying Wang, John Higgins, Allen Templeton, Paul Coleman, Jay Grobler, Ronald Smith, Yunhui Wu

**Affiliations:** 1 Biopharmaceutics, Pharmaceutical Science, Merck & Co., Inc., 770 Sumneytown Pike, West Point, PA 19486, USA; E-Mails: Kimberly_Manser@merck.com (K.M.); Becky_Nissley@merck.com (B.N.); Yunhui_Wu@merck.com (Y.W.); 2 Discovery Pharmaceutical Sciences, Merck & Co., Inc., 770 Sumneytown Pike, West Point, PA 19486, USA; E-Mails: sdclas@pharmasolv.com (S.-D.C.); John_Higgins3@merck.com (J.H.); Allen_Templeton@merck.com (A.T.); 3 Pharmacokinetics, Pharmacodynamics and Drug Metabolism, Merck & Co., Inc., 770 Sumneytown Pike, West Point, PA 19486, USA; E-Mails: Rosa_Sanchez@merck.com (R.I.S.); Michael_Hafey@merck.com (M.H.); Junying_Wang@merck.com (J.W.); 4 Medicinal Chemistry, Merck & Co., Inc., 770 Sumneytown Pike, West Point, PA 19486, USA; E-Mails: Abbas_Walji@merck.com (A.W.); Qun_Dang@merck.com (Q.D.); Jonathan.Bennett@merck.com (D.J.B.); Paul_Coleman@merck.com (P.C.); 5 Process Chemistry, Merck & Co., Inc., 770 Sumneytown Pike, West Point, PA 19486, USA; E-Mail: Jaume_Balsells@merck.com; 6 Toxicology, Merck & Co., Inc., 770 Sumneytown Pike, West Point, PA 19486, USA; E-Mail: Amrithraj_Nair@merck.com; 7 Biology, Merck & Co., Inc., 770 Sumneytown Pike, West Point, PA 19486, USA; E-Mail: Jay_Grobler@merck.com; 8 Formulation Sciences, Merck & Co., Inc., 770 Sumneytown Pike, West Point, PA 19486, USA; E-Mail: Ronald_Smithl@merck.com

**Keywords:** prodrugs, colonic absorption, dog colonic studies, enabling QD dosing, chemistry-enabled drug delivery, enhancing lipophilicity

## Abstract

Prodrugs are chemistry-enabled drug delivery modifications of active molecules designed to enhance their pharmacokinetic, pharmacodynamic and/or biopharmaceutical properties. Ideally, prodrugs are efficiently converted *in vivo*, through chemical or enzymatic transformations, to the active parent molecule. The goal of this work is to enhance the colonic absorption of a drug molecule with a short half-life via a prodrug approach to deliver sustained plasma exposure and enable once daily (QD) dosing. The compound has poor absorption in the colon and by the addition of a promoiety to block the ionization of the molecule as well as increase lipophilicity, the relative colonic absorption increased from 9% to 40% in the retrograde dog colonic model. A combination of acceptable solubility and stability in the gastrointestinal tract (GI) as well as permeability was used to select suitable prodrugs to optimize colonic absorption.

## 1. Introduction

Prodrugs are bioreversible chemistry-enabled modifications of active drugs designed to enhance the pharmacokinetic, pharmacodynamic or pharmaceutical properties of molecules. These chemical modifications improve the drug’s characteristics such as permeability, solubility, chemical or metabolic stability, thus typically enhancing its absorption and bioavailability [1,2,3,4,5,6,7]. A prodrug is ideally pharmacologically inactive and efficiently converted to the active parent through *in vivo* enzymatic and/or chemical transformations. The bioconversion of the prodrug releases the active drug and a promoiety which is preferably physiologically inert and readily eliminated.

For many otherwise efficacious drugs, a short half-life requires more than once daily administration, which increases the probability of non-compliance and therefore less than optimal efficacy. In such cases, strategies employing controlled release or sustained release formulations are used to provide prolonged exposures by delivering a controlled amount of the drug to both the small and large intestine. However, this strategy cannot be used for molecules which do not have the permeability and/or solubility suitable to leverage absorption in the large intestine and thus reduction of the dosing frequency to once a day.

In designing a prodrug that will enhance the absorption of a molecule in the colon, the physiological differences between the large and small intestines must be taken into consideration. Relative to the small intestine, the colon is characterized by low surface area and low aqueous volume. It also presents differences in pH, mucosa, bacteria, and length [8]. Table 1 summarizes some of the differences between the small intestine (the predominant site of absorption of oral drugs) and the colon. Transit time and motility in the colon differ from that in the small intestine and are significantly longer. These are also highly variable, dependent on age, posture, disease, stress, drugs and presence of food. Importantly, colonic fluid volume, which may be 2.5 to 5 fold less than in the small intestine, has a significant impact on colonic absorption. This requires that the compounds have sufficient solubility in the low colonic fluid volume to enable colonic absorption. The longer residence time does provide an opportunity for enhanced absorption should the drug be sufficiently stable, soluble and permeable in the colonic environment. It should be pointed out, however, that the absorption properties of the colon vary along its length from the proximal to the distal colon adding to the complexity of increasing the absorptive properties of molecules. In addition, the main role of the colon is to remove water from the solid matter. The increased solids, low fluid volume, and bacteria in the colon can have a major impact on the colonic absorption of compounds.

**Table 1:** Comparison of the properties of the small and large intestines in humans and dogs [8,9,10,11].

Species	Position	pH	Fluid Volume (mL)	Transit Time (h)
Human	Small intestine (fasted)	6.4–7.5	250	3–4
	Colon	5.5–7.5	50–100	8–24
Dog	Small intestine (fasted)	6.2–6.7	35	2
	Colon	6.4–6.7	0.5	3–12

The pH of the different segments of the gastrointestinal (GI) tract varies, which can also have a significant effect on absorption. From the acidic pH of the stomach (1.5–2 fasted, 2–6 fed) to pH 7.5 in the ileum, the pH generally decreases again to 6.6 in the proximal colon. Vadlamudi *et al*., report that in humans the pH drops to 6.4 in the ascending colon, 6.6 in the mid-colon and finally to 7.0 in the descending colon [8]. Others indicate that the pH varies from 5.5 to 7.0 in the cecum and colon and is 7.0 in the rectum [12,13,14]. The pH of the GI tract is also dependent on disease and the presence of food resulting in potential inter- and intra-subject variability. Compounds that have a pKa in the physiological pH range of 6 to 8 generally prove to be challenging to develop as they are ionic in the GI tract. Ionization usually leads to poorly permeation as the compounds cannot be absorbed through the intestinal wall without the use of active transport mechanisms [15].

An added challenge for colonically-absorbed compounds is that the colonic environment becomes increasingly more viscous and the dissolution rate is significantly reduced leading to inherently slow diffusion through the mucosa. Often when trying to enhance colonic absorption for local colonic delivery, prodrugs use modifications that target the colon’s microbial reductive enzymes, such as sulfasalazine, which undergoes azo-reductive cleavage in the colon [16,17]. Another similar strategy uses glucuronidases, such as with dexamethasone [12,17,18]. Alternatively, gabapentin enacarbil was designed to improve the permeability of gabapentin with addition of a lipophilic promoiety [19]. Gabapentin itself has variable exposure and dose-dependent pharmacokinetics attributed, in part, to its ionization in the GI tract and limited colonic absorption. The gabapentin enacarbil prodrug was designed to block one of the ionization sites of the molecule through the addition of a carbonate lipophilic promoiety [19]. The monocarbonate prodrug is rapidly converted to the active parent in the enterocytes after absorption and enables once-daily dosing where the dosing paradigm was originally restricted to a twice-daily dosing with gabapentin itself.

The goal of this study is to use a similar prodrug strategy as gabapentin and increase colonic absorption of a test drug by increasing lipophilicity. The compound contains a phenolic functional group with pKa of 6.7 and is well absorbed in the small intestine, approximately 80%, but requires twice a day dosing due to its very short half-life and limited colonic bioavailability. Given the physiological pH of the GI tract, it is likely that ionization of the functional group is affecting the permeation and solubility of the compound through-out the GI tract [15,20,21]. An additional challenge is that the dose of the active molecule is significantly higher than for gabapentin, and a prodrug would require optimal solubility while also being more permeable in the colon. However, given the low colonic absorption of the compound, increases in colonic permeation were considered paramount. A preclinical dog colonic absorption model was used to initially evaluate the enhancement of colonic permeation of the prodrugs. Published literature indicates that, in general, the dog model is a good model to predict human colonic absorption [22,23,24]. Sutton *et al*., shows a good relationship of relative bioavailability in dogs and human following oral and colonic administration for a relatively large number of compounds [24]. Overall dogs can be considered reasonable predictors of colonic absorption in humans.

## 2. Experimental

### 2.1. Materials

The prodrugs were provided by Merck & Co., Ltd. (West Point, PA, USA) and used as received (>95% purity). Captisol^®^ was purchased from Ligand (La Jolla, CA, USA). Tween 80 (Polysorbate 80) and solvents [high-performance liquid chromatography (HPLC) grade] were obtained from Fisher Scientific (Pittsburgh, PA, USA). Simulated Gastric Fluid (SGF) or Fasted State Simulated Intestinal Fluid (FaSSIF) was made from SIF powder (Biorelevant, Cryodon, Surrey, UK).

### 2.2. Physicochemical Characterization

#### 2.2.1. Solubility in Biorelevant Media

Solubility measurements were carried out by suspending excess solids (up to 1 mg/mL) in SGF or FaSSIF [25] and stirred at room temperature (22 °C). The excess solids were isolated by centrifugation at 10,000 relative centrifugal force at 37 °C for 10 min. The solubility and stability of the prodrug in the supernatant was determined by reverse-phase HPLC using an Ascentis Fused-Core C-18 column (4.6 × 100 mm, 2.7 µm particle) at 40 °C (Sigma-Aldrich, St. Louis, MO, USA). With a gradient mobile phase of 10%–95% acetonitrile in 6 min in 0.1% phosphoric acid in water followed by a hold at 95% acetonitrile for 2 min. The flow rate was 1.8 mL/min and run times were 8 min with 2 min for re-equilibration. Injection volumes were 2–5 μL and detection was at 210 nm.

#### 2.2.2. Stability in Biorelevant Media

The compounds were suspended in SGF or FaSSIF for 1 or 5 h, then dissolved in 50/50 acetonitrile/water and analyzed by reverse-phase HPLC using the method described above.

### 2.3. *In Vitro* Permeability

The *in vitro* permeation studies are similar to those described elsewhere [26]. Briefly, LLC-PK1 cells were cultured in 96-well transwell culture plates. The area of membrane was 0.11 cm^2^. The prodrugs (final concentration 1 μM) were prepared in Hank’s Balanced Salt Solution (HBSS) with 10 mM HEPES. Substrate solution (150 μL) was added to either the apical (A) or the basolateral (B) compartment of the culture plate, and buffer (150 μL; +10 mM HEPES) was added to the compartment opposite to that containing the compound. At t = 3 h, 50 μL of sample was taken out from both sides and analyzed by LC-MS/MS for both parent drug and prodrug. Verapamil (1 μM) was used as the positive control. The experiment was performed in triplicate. The reported apparent permeation (P_app_) is the average of the P_app_ for transport from A to B and P_app_ for transport from B to A at t = 3 h and is expressed as 10^−6^ cm/s.

### 2.4. Pharmacokinetic Procedures

Male Beagle Dogs (Marshall Farms, North Rose, NY, USA) weighing between 8.0–13 kg were used for the *in vivo* studies. Merck is dedicated to the ethical and responsible treatment of all animals used in the development of medicines and vaccines. All animal procedures were done in accordance with guidelines from the Institutional Animal Care and Use Committee at Merck. Following overnight-fasting, dogs were dosed either orally or colonically via a retrograde catheter method with drug solution formulation at doses between 0.5 to 4 mpk of compound with a dosing volume of 1 ml/kg. Oral dosing was accomplished via oral gavage immediately followed by 5 mL rinse via the same 18 French oral gavage tube (Tyco Healthcare Group LP, Mansfield, MA, USA). Colonic dosing was achieved with a lubricated 7French catheter (Covidien, LLC, Mansfield, MA, USA) that was inserted through the anal sphincter and carefully advanced to a distance of 20 cm (descending colon). Dogs were repositioned if needed to aid in catheter advancement. Once the catheter was positioned, the formulation was administered as a bolus dose via a 20 mL syringe attached to the catheter via 2-way valve which was turned post-dose allowing the catheter to be flushed with 5 mL of water delivered via another syringe. This ensured complete delivery of the formulation. Water was restricted for 1 h post dose. Food was returned at 4 h after dosing. Blood (1 mL) was drawn at pre-dose, 0.25, 0.5, 1, 2, 4, 6, 8 and 24 h post-dosing. The plasma was separated by centrifugation (10 minat 2,500 g) and kept frozen at –70 °C until analysis by LC-MS/MS. LC-MS/MS analysis was done using a Transcend LX2 Multiplexed UPLC with Rheos Allegro quaternary pumps (Thermo, CTC, Pittsburgh, PA, USA) interfaced to the API-4000 or API-5000 mass spectrometer (Life Technologies, Carlsbad, CA, USA) utilizing the turbo ion spray interface and a Waters XSELECT HSS T3 XP column (50 × 2.1 × 2.5 µm) (Milford, MA, USA). The concentrations of the drug and prodrugs in dog plasma were determined using a non-validated LC-MS/MS assay following a protein precipitation extraction and addition of an appropriate internal standard. Quantification was done by monitoring transitions specific for each molecule. The methods were linear across a concentration range of 2 or 5 to 5,000 nM.

## 3. Results and Discussion

The active parent compound consists of a phenolic functional group with a pKa of 6.7. Due to the ionization of the functional group, the permeability and solubility are affected through-out the GI tract. From Table 2 it can be seen that the parent compound has poor colonic absorption which could be, in part, attributed to its pKa and ionization in the colon. The current prodrug effort was initiated to evaluate the feasibility of increasing the absorption window of the parent compound. The strategy used to enhance colonic absorption and potentially enable QD dosing was to select promoieties that would block the ionization and increase the lipophilicity of the compound. The initial screening for prodrug viability was based on solubility, stability and prodrug permeation. If the prodrug met the minimum requirements on solubility and stability and showed bioconversion to parent *in vivo* (*i.e*., via rat and/or dog) the compound was advanced to a dog colonic absorption study in which the colonic absorption/permeation of the prodrug was determined. Each iteration of prodrug design was aimed to probe one of the various physicochemical aspects that could potentially affect colonic absorption (e.g., solubility, permeability, stability, *etc*.).

Initially, more than 60 prodrugs were synthesized and screened for stability in biorelevant media (SGF and FaSSIF). Those with chemical stability greater than 90% were selected for further evaluation of solubility in biorelevant media, *in vitro* permeability and colonic absorption. As seen in Table 2, evaluation of the physicochemical properties of the prodrugs alone did not immediately indicate a best-in-class molecule, rather the physicochemical properties of the prodrugs need to be considered in conjunction with the colonic absorption data. In the dog colonic absorption studies the prodrug was dosed and the absorption of both the prodrug and parent were evaluated. The absorption is reported in terms of parent exposure unless otherwise indicated.

**Figure 1:**
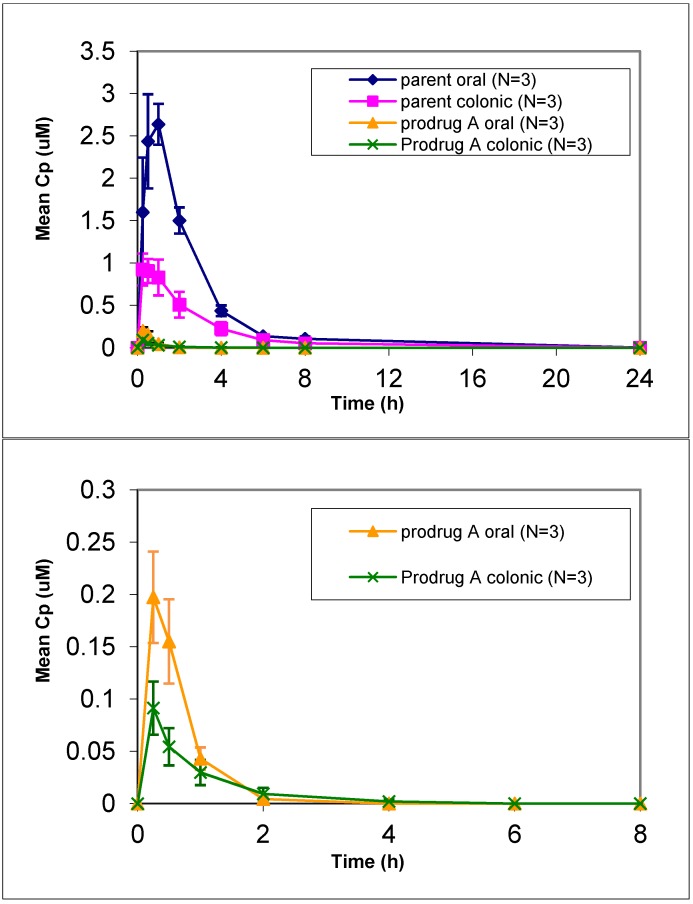
Mean plasma concentration *vs*. time profiles of prodrug A and parent following oral and colonic administration of prodrug solution to fasted Beagle dogs at a dose of 4 mpk (mean ± SE). [bottom graph is zoom in for prodrug only profile].

Prodrug A had one of the highest solubilities of the prodrugs in both SGF and FaSSIF, however slight degradation of prodrug A was seen in these media. *In vivo* dog studies did show efficient prodrug conversion (>80%) with less than 2% prodrug circulating in plasma after oral or colonic administration (Figure 1). Prodrug A showed an increased lipophilicity compared to the parent but was similar to the other prodrugs (Table 2). Given the increase in lipophilicity it was surprising to see a lower LLC-PK1 permeation for prodrug A. Despite the lower permeation predicted via the LLC-PK1 cell assay, administration of a solution of prodrug A resulted in a 40% relative colonic bioavailability. This represented a 4-fold improvement in relative colonic bioavailability compared to the parent, which gave only 9% relative colonic bioavailability. However prodrug A also showed a decrease in oral absorption compared to the parent, so while the colonic absorption was increased 2.5-fold, the oral absorption was decreased by 1.5-fold leading to a potentially inflated relative colonic absorption value. This suggests that while the colonic absorption was increased for prodrug A, compared to parent the overall oral absorption of prodrug A seems to be lower than desired. Given the lower stability in biorelevant media of prodrug A compared to the other prodrugs, the importance of stability was considered.

**Table 2:** Physicochemical properties of the prodrug molecules compared to that of the parent drug molecule in terms of solubility in simulated gastric fluid (SGF), simulated fasted state intestinal fluid (FaSSIF), permeation values from LLC-PK1 assay (with cyclosporine A), calculated partition coefficient (ALogP 98) and dog colonic absorption.

Compound	LLC-PK1 P_app_ (×10^-6^ cm/s)	Stability in SGF ^1^ (1 h)% Claim	Stability in FaSSIF ^2^ (5 h)% Claim	Solubility in SGF (mg/mL) 1 h	Solubility FaSSIF (mg/mL) 1h	ALogP 98 ^3^	Percent Relative Dog Colonic Absorption ^4^
Parent	11.6	98.36%	99.40%	0.01	0.50	−0.7	9%
Prodrug A 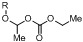	5.8	93.80%	90.97%	0.37	0.33	0.9	40%
Prodrug B 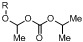	8.9	100.02%	99.92%	0.02	0.03	1.3	43%
Prodrug C 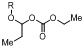	11.9	99.32%	98.27%	0.04	0.25	1.5	31%
Prodrug D 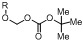	11.9	100.14%	101.61%	0.06	0.06	1.3	30%
Prodrug E 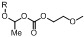	1.7	95.50%	96.27%	6.6	0.60	0.4	5%
Prodrug F 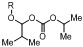	15.4	91.32%	98.45%	0.02	0.04	2.2	10%

^1^ SGF: simulated gastric fluid; ^2^ FaSSIF: Fasted state simulated intestinal fluid; ^3^ The octanol/water partition coefficient (ALogP 98) was calculated using Accelrys Cerius2 Software (Accelrys, Inc., San Diego, CA, USA);^4^ Ratio of AUC_0-24 h_ colonic to AUC_0-24 h_ oral administration.

Comparing physicochemical properties of prodrugs A through D, prodrugs B, C and D had lower solubility than prodrug A but were more stable in the *in vitro* biorelevant media stability assays. More stable prodrugs could lead to better colonic absorption. Prodrugs B, C and D had permeation values comparable to the parent and slightly higher than prodrug A (Table 2). The relative colonic bioavailability for the parent when dosing prodrugs B, C or D was 43%, 31% and 30%, respectively. Prodrugs B and C showed a higher relative colonic absorption ratio compared to the parent but lower oral absorption compared to parent and similar to lower colonic absorption (Table 3). With this significant drop in oral absorption from prodrugs B and C and only moderate increase in colonic absorption, it is difficult to say that the overall colonic absorption is increased in prodrugs B and C compared to parent. Prodrug D, given the same increased *in vitro* stability in biological media, showed similar relative colonic absorption to prodrugs A, B, and C but with overall better oral absorption. Compared to parent, prodrug D gave similar oral absorption and showed a 2.5-fold increase in colonic absorption. This increase in colonic absorption was comparable to what was seen with prodrug A but with no loss in oral absorption suggesting that prodrug D had generally better colonic absorption than parent based on the dog colonic studies.

**Table 3:** Mean [± SE] pharmacokinetic parameters for parent after oral and colonic administration of solutions of the prodrug to fasted Beagle dogs ^1^.

CompoundDosed (vehicle)	Dose (mpk)	DosingRoute	nAUC_0-24 h_^2^ (µM h/mpk)	nC_max_^2^(µM/mpk)	T_max_^3^ (h)	Percent Relative Dog Colonic Absorption
Parent (3% Tween)	4	Oral	2.92 ± 0.48	1.40 ± 0.25	0.5(0.3–0.5)	-
		Colonic	0.30 ± 0.26	0.06 ± 0.04	0.5(0.3–2.0)	9%
Prodrug A (3% Tween)	4	Oral	1.91 ± 0.12	0.69 ± 0.09	1.0(0.5–1.0)	-
		Colonic	0.76 ± 0.21	0.24 ± 0.04	0.5(0.3–1.0)	39%
Prodrug B (10% Tween)	1	Oral	0.94 ± 0.05	0.38 ± 0.03	1(0.5–1)	-
		Colonic	0.40 ± 0.13	0.09 ± 0.02	1	43%
Prodrug C (10% Tween)	1	Oral	0.77 ± 0.13	0.27 ± 0.05	0.25	-
		Colonic	0.24 ± 0.04	0.06 ± 0.01	1(0–1)	31%
Prodrug D (30% Captisol^®^)	4	Oral	2.4 ± 0.14	0.83 ± 0.21	1.0(0.5–2.0)	-
		Colonic	0.72 ± 0.07	0.17 ± 0.01	1.0	30%
Prodrug E (10% Tween)	0.7	Oral	4.35 ± 1.3	1.65 ± 0.35	0.5(0.5–1)	-
		Colonic	0.24 ± 0.15	0.06 ± 0.02	0.5	5%
Prodrug F (10% Tween)	1	Oral	0.75 ± 0.02	0.26 ± 0.01	1 (0.5–1)	-
		Colonic	0.07 ± 0.13	0.02 ± 0.01	2 (0.5–2)	10%

^1^ It should be noted that the plasma exposure for the prodrugs were below the limit of quantitation at 2 h for oral and colonic dosing; ^2^ nAUC_0-24 h_ and nC_max_ refer to dose normalized values; ^3^ For T_max_, median value is provided.

Overall this data indicate that for this series, prodrug stability *in vitro* was generally not a significant factor as long as the greater than 90% stability criteria in biological media was met. However the potential contributing effect of solubility *in vivo* cannot be ruled out given the high solubilizing nature of the dosing vehicles of these prodrugs. It should also be noted that prodrug D was dosed as a Captisol^®^ solution rather than in Tween. This was a result of the lower initial prodrug solubility in vehicle.

To further investigate the effects of solubility on the colonic absorption of the prodrugs, prodrug E was evaluated. Prodrug E was found to have slight degradation in biological media compared to prodrugs B, C and D but not significantly different than the degradation seen with prodrug A. Prodrug E did, however, show solubility in SGF that was significantly higher than that of the other prodrugs (18 to 330 fold increase) and in a similar trend a 2 to 20 fold higher FaSSIF solubility (Table 2). For this reason prodrug E was used to assess the relationship between increased prodrug solubility and colonic absorption. The results from prodrug E show that compared to the other prodrugs there was an increase in oral absorption but a low colonic absorption resulting in an overall relative bioavailability for colonic administration of only 5% for prodrug E (Table 3). This was analogous to the relative colonic absorption seen with the parent itself. For prodrug E the colonic absorption was similar to prodrug C and lower than that of the prodrugs A, B and D but the oral absorption was increased significantly compared to parent and that of all the prodrugs (1.5-fold increase compared to the parent and 1.8 to 5.6 fold increase compared to the other prodrugs). This increase in oral absorption but stagnant colonic absorption suggests that the overall absorption was increased from prodrug E but the colonic absorption was not. To further support this, the ALogP value and LLC-PK1 permeation of prodrug E were considerably lower than those of the previous prodrugs (Table 2) implying that colonic bioavailability was strongly influenced by permeation and increases in solubility alone were not sufficient to increase colonic absorption for this series of prodrugs. An important consideration for absorption with prodrugs, and non-prodrug small molecules alike, is the interplay of solubility and permeation. To this end, prodrug F was used to assess the understanding between cell culture P_app_ values and the colonic permeation seen in the dog colonic model. Prodrug F had a higher lipophilicity and LLC-PK1 permeation than all the other prodrugs with similar biorelevant stability but its solubility in biorelevant media was also one of the lowest (Table 2). The dog colonic absorption results from prodrug F show that the relative colonic absorption for parent when dosing prodrug F was only 10% (Table 3). The oral dosing of prodrug F showed similar exposure compared to prodrug C and lower exposure than from dosing of prodrugs A, B, D and E. Prodrug F did give a significantly lower colonic exposure compared to all prodrugs and parent (3 to 11 fold lower colonic absorption). The lower colonic exposure is likely not from dosing issues as the observed oral exposure of parent was similar to prodrug C. Levels of prodrug F in plasma did seem to be higher with colonic dosing than for the other prodrugs (data not shown) but in this case this did not translate into higher parent exposure. The significantly lower colonic absorption for prodrug F could be attributed to differences in metabolic profile and/or *in vivo* bioconversion. Overall this data suggests a good correlation of LLC-PK1 permeation and ALogP 98 values for the prodrugs which was not surprising, however these studies also indicated that the permeation of the prodrugs seen from the LLC-PK1 permeation assay did not correlate well with dog colonic absorption. Thus other factors in addition to permeation seem to affect colonic absorption for these prodrugs. This could potentially be vehicle related, as the dog PK studies were evaluated using different vehicles (Table 3). In addition the 3-fold difference in LLC-PK1 P_app_ values between the prodrugs tested may not be sufficient (without solubility enhancement) to increase colonic permeation.

The formulation of a compound also plays an important role in determining colonic absorption in relation to potential sustained release feasibility in the clinic, so it is always important to take the dosing formulation into account for colonic absorption studies. Overall the higher the percentage of solubilizing agent, the lower the feasibility of being able to achieve similar levels of absorption from a solid dosage form in the clinic. For the dog colonic absorption studies, the prodrugs were generally formulated as solutions with high amounts of surfactant (*i.e*., 10% Tween), levels which are higher than the typical surfactant amount in clinical dosage forms (*i.e*., 0.5% surfactant). This suggests that due to enhanced solubilization the colonic absorption reported may be an overestimation of what can be achieved with a clinical dosage form. Despite the limited translatability of these studies to the clinic, these results are useful to gauge the impact of permeability and solubility on the colonic absorption. It should be noted that while compound phase and *in vivo* bioconversion of the prodrugs were not highlighted in this article, these are important aspects to the overall success of a prodrug strategy and were extensively evaluated during these studies (manuscripts in preparation).

## 4. Conclusions

These studies show that a balance of increases in lipophilicity and solubility were necessary to enhance the colonic permeability of the prodrugs. If the balance was skewed too far in one direction, colonic absorption was negatively affected. The relative colonic absorption for the parent from prodrug dosing was between 30%–43% for most of the prodrugs relating a 3-to-4 fold higher relative bioavailability for colonic absorption compared to the parent, which gave a relative colonic bioavailability of only 9%. The exceptions were prodrugs E and F which gave relative colonic absorptions similar to parent at 5% and 10%, respectively. The low colonic absorption of prodrugs E and F was most likely linked to their specific physicochemical or metabolic properties.

Critically considering the changes in oral and colonic absorption during these studies, prodrug D gave a similar oral absorption as parent and a 2.5-fold increase in colonic absorption therefore likely represented the overall best increase in colonic absorption for the prodrugs. However, it should be noted that for the dog colonic absorption studies the prodrugs were formulated in highly solubilizing vehicles (*i.e*., 10% Tween, 30% Captisol^®^). These dosing vehicles represent an extremely high amount of solubilizing agent compared to the typical clinical formulation and may represent an overestimation of the colonic absorption. Overall this research shows prodrugs have been made that exhibit a higher relative colonic bioavailability of the parent than dosing of parent alone. When formulated as a sustained release formulation, it might be expected that a suitable prodrug could provide a positive step to improving colonic absorption and dosing regimen of the parent.
